# The Damaging Outcome of the POLAR Phase III Trials Was Due to Avoidable Time-Dependent Redox Interaction between Oxaliplatin and PledOx

**DOI:** 10.3390/antiox10121937

**Published:** 2021-12-03

**Authors:** Jan Olof G. Karlsson, Per Jynge, Louis J. Ignarro

**Affiliations:** 1 Department of Biomedical and Clinical Sciences, Division of Clinical Chemistry and Pharmacology, Linköping University, 581 83 Linköping, Sweden; 2 Department of Radiology, Innlandet Trust Hospital, Gjøvik Hospital, 2819 Gjøvik, Norway; per.jynge.ha@gmail.com; 3 Department of Pharmacology, UCLA School of Medicine, 264 El Camino Drive, Beverly Hills, CA 90212, USA; lignarro@gmail.com

**Keywords:** calmangafodipir, chemotherapy-induced peripheral neuropathy, colorectal cancer, drug interactions, mangafodipir, oxaliplatin

## Abstract

On 2 July 2021, highly negative results were reported from the POLAR A and M phase III trials in patients with colorectal cancer, treated with an oxaliplatin-based regimen and co-treated with calmangafodipir (CaM; PledOx^®^; PledPharma AB/Egetis Therapeutics AB) or placebo. The results revealed persistent chemotherapy-induced peripheral neuropathy (CIPN) in 54.8% of the patients treated with PledOx, compared with 40.0% of the patients treated with the placebo (*p* < 0.05), i.e., a 37% increase in incidence of the side effect that the trial was aimed to prevent. The damaging outcome of the trials differed diametrically from an in-parallel conducted mice study and from a clinical trial with mangafodipir, the active ingredient of CaM. According to the authors of the POLAR report, the etiology of the profound increase in CIPN in the PledOx arm is unclear. However, these devastating effects are presumably explained by intravenous administrations of PledOx and oxaliplatin being too close in time and, thereby, causing unfavorable redox interactions between Mn^2+^ and Pt^2−^. In the mice study as well as in the preceding phase II clinical trial (PLIANT), PledOx was administered 10 min before the start of the oxaliplatin infusion; this was clearly an administration procedure, where the devastating interactions between PledOx and oxaliplatin could be avoided. However, when it comes to the POLAR trials, PledOx was administered, for incomprehensible reasons, “on Top of Modified FOLFOX6” at day one, i.e., after the two-hour oxaliplatin infusion instead of before oxaliplatin. This is a time point when the plasma concentration of oxaliplatin and Pt^2+^-metabolites is at its highest, and where the risk of devastating redox interactions between PledOx and oxaliplatin, in turn, is at its highest.

This opinion article deals with the harmful redox-based interactions between two intravenously administered metallo-therapeutic agents—cytotoxic oxaliplatin and cytoprotective calmangafodipir [CaM; MnCa_4_(DPDP)_5_]—and addresses the issue of how such interactions can be avoided in combination therapy, with particular reference to the POLAR A and M trials (ClinicalTrials.gov Identifier NCT40343555 and NCT03654729).

On 7 July 2020, Canta and co-workers reported in *Antioxidants* [1] that CaM, a stabilized form of mangafodipir (MnDPDP) [2], reduced sensory alterations and prevented intra-epidermal nerve fiber loss in a mouse model of chemotherapy-induced peripheral neuropathy (CIPN). CIPN is a most troublesome and dose-limiting side effect of the anticancer agent oxaliplatin, and these promising results with CaM in mice were in accordance with relevant mangafodipir experience with humans [3,4]. Recent clinical studies, however, have reported highly negative results of CaM (PledOx^®^, PledPharmaAB/Egetis Therapeutics AB) [5]. Thus, there is a need to discuss the etiology of seemingly opposite effects of mangafodipir and CaM. That is, why mangafodipir prevents/cures CIPN, whereas CaM seemingly exacerbates this condition.

The recent POLAR A and M trials in patients with colorectal cancer (CRC), treated with an oxaliplatin-based regimen, were prematurely closed on 6 April 2020 due to a significantly higher number (14 versus 2) of platinum-related hypersensitivity reactions (HSR) in the PledOx arms, versus the placebo [5]. The closure occurred after a little more than 400 out of the 700 CRC patients had received 6 or more cycles of chemotherapy. The main results, as presented on 2 July 2021, in a congress abstract by Qvortrup, Pfeiffer et al., revealed persistent CIPN in 54.8% of the patients treated with PledOx compared with 40.0% of the patients treated with placebo (*p* < 0.05) , i.e., a 37% increase in incidence of side effects that the trial was aimed to prevent—a highly damaging result.

The outcome of the POLAR A and M trials differed diametrically from the positive outcome in cancer patients, reported by Coriat and co-workers in 2014 [3]. In that study, the MRI contrast agent and catalytic antioxidant, mangafodipir (Teslascan™; GE Healthcare), relieved and even reversed oxaliplatin-associated CIPN. The Coriat results also confirmed a previous case report by Yri et al. [4], in which a CRC patient, co-administered with Teslascan during 14 cycles of oxaliplatin, did not show any sign of CIPN, except during one cycle (the 7th out of totally 15 cycles), when Teslascan was deliberately omitted.

Prior to the POLAR studies Glimelius and co-workers, on 15 November 2017, reported from a phase II clinical trial (PLIANT) [6], which was investigating the efficacy of PledOx in CRC patients going through oxaliplatin-based chemotherapy. These authors claimed that PledOx was effective against persistent/chronic CIPN and recommended going into phase III. Karlsson and Jynge disagreed and warned against proceeding into phase III without a strong enough scientific proof of efficacy [7,8], whereas Glimelius et al. maintained that their data were “trustworthy” enough [9]. Importantly, there was nothing in their data indicating that PledOx might exacerbate CIPN. Instead, these suggested a positive effect, which was, according to Karlsson and Jynge [7], far too weak for going into expensive phase III trials. The main problem with the PLIANT study was its extremely low frequency of oxaliplatin-induced side effects in the placebo group. 

According to the authors of the POLAR report [5], the etiology of the profound increase in CIPN, as well as HSR, in the PledOx arms, is unclear. However, these damaging effects can be explained by intravenous administrations of PledOx and oxaliplatin being too close in time and thereby causing unfavorable, metal-based redox interactions between these two drugs. Thus, combining a Mn^2+^- and a Pt^2+^-containing agent, where the latter cation has a considerably higher reduction potential than the former, may lead to oxidation of the former. Accordingly, electron paramagnetic resonance (EPR)-guided competition experiments between mangafodipir and K_2_PtCl_4_ indicate that Pt^2+^ may drive oxidation of Mn^2+^ [10]. The oxidation product Mn^4+^ may subsequently drive cellular oxidative/nitrosative stress (ONS) through tyrosine nitration, causing irreversible inactivation of key proteins like the mitochondrial antioxidant enzyme manganese superoxide dismutase (MnSOD) [11]. Mitochondrial cytochrome c is another target for tyrosine nitration that triggers a conformational change in this protein, resulting in an alternative conformation lacking its normal electron transport capacities [11].

In the cited mouse study [1], PledOx was administered 10 min ahead of oxaliplatin, similar to the time point in the PLIANT trial [6], (ClinicalTrials.gov Identifier: NCT01619423), seemingly an administration procedure where interactions between PledOx and oxaliplatin could be avoided or made less apparent. Unfortunately, the exact time point for administration in POLAR A and M is neither given at ClinicalTrials.gov nor in the report [5]. However, it appears that PledOx, for incomprehensible reasons, was administered “on Top of Modified FOLFOX6” at day one (ClinicalTrials.gov Identifier: NCT04034355 and NCT03654729), i.e., after the two-hour oxaliplatin infusion instead of ahead of oxaliplatin. This is a time point when the plasma concentration of oxaliplatin and Pt^2+^-metabolites is at its highest, and hence where the risk of devastating redox interactions between Mn^2+^ and Pt^2+^ is at its highest. This presumably explains the disastrous results of the POLAR trials. We have repeatedly, but without success, asked the first and last authors of the POLAR report [5]—Qvortrup from the Copenhagen University and Pfeiffer from the University of Southern Denmark, respectively—to confirm our interpretation of the time point for PledOx administration.

Even in the lack of the exact time point for administration, it appears relevant to ask why Coriat and co-workers [3] achieved such positive effects with mangafodipir, the active ingredient of PledOx. Intriguingly, an explanation may be found in the formulations used for delivery of the active ingredients and the content of additives. Thus, the formulations used for delivery of mangafodipir in the Coriat study, and that used in the PLIANT and POLAR trials, differ from each other; this is because the ready-to-use Teslascan, but presumably not PledOx, contains 6 mM ascorbic acid. In Teslascan, the ascorbic acid is added to avoid oxidation of Mn^2+^ and subsequent loss of paramagnetic strength when used as an MRI contrast agent. Therefore, there are good reasons to anticipate that ascorbic acid not only protects Mn^2+^ from oxidation during storage but also during in vivo infusion of Teslascan, immediately after oxaliplatin, in a chaperone manner, as applied by Coriat. However, with calcium-containing formulations, like that of PledOx, ascorbic acid cannot be included since it easily degrades into oxalate [12], thereby forming insoluble calcium oxalate. Another contributing factor to the different outcome of Teslascan compared with PledOx might be that the former was administered at an infusion rate about 6 times lower than the latter (30 versus 5 min infusions).

The capability of mangafodipir to combat acute ONS is mainly due to its MnSOD mimetic activity [2]. However, its efficacy to combat persistent/chronic oxaliplatin-associated CIPN, presumably caused by long lasting body retention of Pt^2+^, requires a different mechanism of action [10]. EPR-guided competition experiments between mangafodipir versus Pt^2+^, and other divalent cations, suggest that fodipir binds Pt^2+^ with high enough affinity to act as a chelation drug [10], i.e., a drug that binds the metal cation in question and facilitates its mobilization and subsequent renal excretion from the body.

Furthermore, at the time for closure of the POLAR trials, the FDA had put the study on temporary hold due to possible CNS-related adverse events. In case such events were caused by PledOx as obviously suspected by FDA, experimental data support that both Mn^2+^ and Mn^3+^ may induce oxidative stress, but Mn^3+^ is an order of magnitude more potent than Mn^2+^ [13]. Furthermore, uptake of manganese into the brain is much higher for Mn^3+^ than for Mn^2+^ [14]. It is hence possible that Mn^2+^ derived from PledOx after oxidation by Pt^2+^, in addition, may increase the risk of typically manganese-related CNS symptoms.

In vivo mixing of two metal-complexes with inherent redox properties may lead to devastating drug interactions. A costly lesson to be learned from POLAR trials, for both participating patients and shareholders, is that PledOx (CaM) should not be used in combination with platinum-containing drugs, such as oxaliplatin and cisplatin, without a considerable time interval between their administrations. When it comes to Teslascan and its curing effect on oxaliplatin-associated chronic CIPN, manganese is probably not needed, and from a safety perspective, the manganese-free chelator fodipir seems to be a better alternative than its manganese-containing counterpart. However, in order to lessen acute adverse effects caused by non-platinum chemotherapy, including, for example, neutropenia, mucositis and cardiac failure, little or nothing speaks against pretreatment with PledOx, as an effective and safe method.

A more general lesson to be learned is that unexplored combinations of drugs may elicit harmful interactions. Such interactions may not necessarily give insight into the toxicity of the individual drugs, per se. The risk of unfavorable drug interactions is something that has to be carefully scrutinized before exposing a larger phase III population to the actual drug combination.

As founders of PledPharma, including also the first inventor of CaM, the authors of the current opinion article fear that poor pharmacological/pharmaceutical performance of PledPharma/Egetis will erase calmangafodipir/mangafodipir from the list of promising drug candidates with a wide range of unique indications.
